# Conservation and Identity Selection of Cationic Residues Flanking the Hydrophobic Regions in Intermediate Filament Superfamily

**DOI:** 10.3389/fchem.2021.752630

**Published:** 2021-09-02

**Authors:** Wenbo Zhang, Mingwei Liu, Robert L. Dupont, Kai Huang, Lanlan Yu, Shuli Liu, Xiaoguang Wang, Chenxuan Wang

**Affiliations:** ^1^ State Key Laboratory of Medical Molecular Biology, School of Basic Medicine Peking Union Medical College, Institute of Basic Medical Sciences Chinese Academy of Medical Sciences, Beijing, China; ^2^ William G. Lowrie Department of Chemical and Biomolecular Engineering, The Ohio State University, Columbus, OH, United States; ^3^ Shenzhen Bay Laboratory, Shenzhen, China; ^4^ Department of Clinical Laboratory, Peking University Civil Aviation School of Clinical Medicine, Beijing, China; ^5^ Sustainability Institute, The Ohio State University, Columbus, OH, United States

**Keywords:** protein assembly, coiled-coil, self-assembly, hydrophobic interactions, charge-related interactions

## Abstract

The interplay between the hydrophobic interactions generated by the nonpolar region and the proximal functional groups within nanometers of the nonpolar region offers a promising strategy to manipulate the intermolecular hydrophobic attractions in an artificial molecule system, but the outcomes of such modulations in the building of a native protein architecture remain unclear. Here we focus on the intermediate filament (IF) coiled-coil superfamily to assess the conservation of positively charged residue identity via a biostatistical approach. By screening the disease-correlated mutations throughout the IF superfamily, 10 distinct hotspots where a cation-to-cation substitution is associated with a pathogenic syndrome have been identified. The analysis of the local chemical context surrounding the hotspots revealed that the cationic diversity depends on their separation distance to the hydrophobic domain. The nearby cationic residues flanking the hydrophobic domain of a helix (separation <1 nm) are relatively conserved in evolution. In contrast, the cationic residues that are not adjacent to the hydrophobic domain (separation >1 nm) tolerate higher levels of variation and replaceability. We attribute this bias in the conservation degree of the cationic residue identity to reflect the interplay between the proximal cations and the hydrophobic interactions.

## Introduction

**FIGURE 1:**
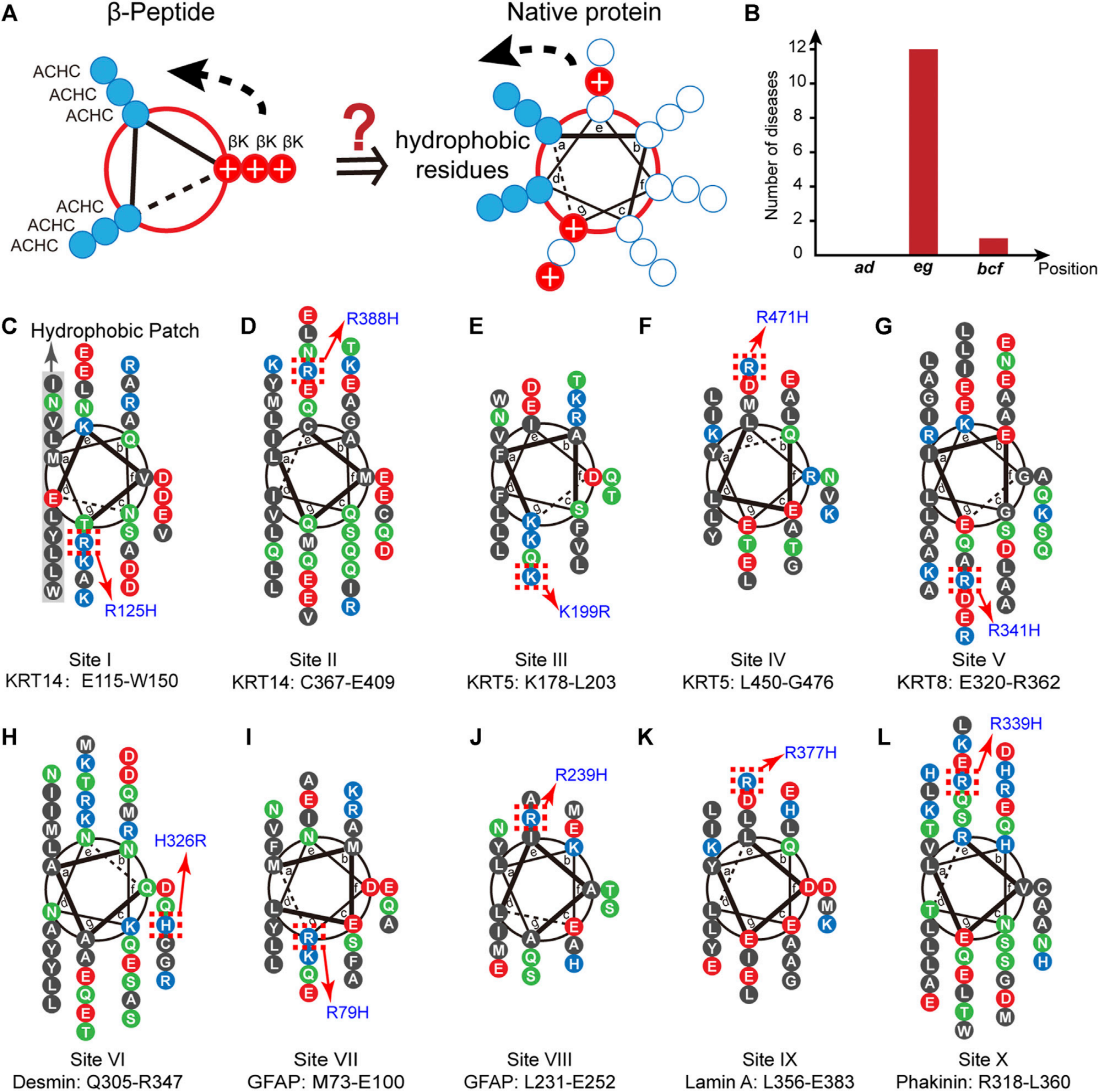
The conservation of proximal cationic side chain in the IF superfamily. **(A)** The helix-wheel diagrams of a globally amphiphilic β^3^-peptide **(left)** and native α-helical peptide **(right)**. The solid blue discs represent nonpolar residues and the solid red discs with a white plus sign represent the side chains of positively charged residues. (**B**) The distribution of the number of diseases associated with single cation-to-cation substitution. **(C–L)** Helix-wheel diagrams of the coil 1A domains in KRT14 (**C, D**, site I and II) in the type I family, KRT5 (**E, F**, site III and IV) and KRT8 (**G**, site V) in the type II family, Desmin (**H**, site VI) and GFAP (**I, J**, site VII and VIII) in the type III family, Lamin A (**K**, site IX) in the type V family, and phakinin (**L**, site X) in the type VI family. Blue, cationic residues; red, anionic residues; black, nonpolar residues; green, polar and uncharged residues. The hotspots in the homology sequences are denoted by red dotted boxes.

The complex networks of weak noncovalent interactions, *i.e.*, hydrophobic interactions, electrostatic interactions, van der Waals interactions, and hydrogen bonding, engender the highly specific folding and intermolecular recognition of proteins (Pace et al., 2014; Zhang et al., 2020a; Zhang et al., 2020b). To date, however, a *de novo* prediction of the tertiary and quaternary structures from an ensemble of amino acid scratches via a free energy summation approach remains challenging (Rego et al., 2019; Zhang et al., 2020a). This difficulty is further exacerbated by the nonadditive interplay among the various noncovalent interactions. Additionally, conceptual models regarding the different types of noncovalent interactions as divided elements are typically inadequate (Giovambattista et al., 2007; Huang et al., 2015; Ma et al., 2015; Wang et al., 2017). Recently, we explored how the identity of an adjacent cation modulates the strength of hydrophobic interactions generated by a nonpolar domain in an artificial system, specifically in synthetic amphiphilic β-amino acid oligomers (Ma et al., 2015; Wang et al., 2017). The amphiphilic β-amino acid oligomers adopt a 14-helical conformation and flank the positively charged residues of either β^3^-homolysine or β^3^-homoarginine in the proximity of a nonpolar domain formed by six cyclohexyl groups (from *trans*-2-aminocyclohexanecarboxylic acid (ACHC) residues) (Figure 1A) (Ma et al., 2015; Wang et al., 2017). A single-molecule force experiment revealed that the side chains of homolysine and homoarginine display divergent effects on the magnitude of the hydrophobic interactions mediated by the ACHC-rich domain. Specifically, the protonation of β^3^-homolysine strengthens hydrophobic interactions, whereas the protonated β^3^-homoarginine diminishes hydrophobic interactions (Ma et al., 2015). This pronounced modulation is attributed to the distinct impacts of the charged groups on the local density and organizational structure of interfacial water (Wang et al., 2017). A key question raised from this study is whether or not this striking outcome affects a biologically relevant system. Considering the prevalence of cationic-hydrophobic heterozygotes in nature, such as cell-penetrating peptides (e.g., melittin and gramicidin) and coiled-coil architectures, we are motivated to investigate whether the bias of the neighboring cation identity at the boundary of a nonpolar domain is a broadly distributed evolutionary selection tool that exists in native protein systems.

We chose the superfamily of intermediate filament (IF)-forming proteins as a model native system to evaluate the conservation of positively charged residues in the proximity of nonpolar patches in the IF members via a biostatistical approach. The IF superfamily comprises six multigene families ranging from type I to type VI (Omary et al., 2004). All IF-forming proteins feature a central α-helical rod domain comprising four heptads of repeating helical segments (1A, 1B, 2A, and 2B) that present a hydrophobic central core that provides the driving force for the formation of twisted coiled-coils that further build up the structural skeletons accounting for cell mechanics (Omary et al., 2004; Jacob et al., 2018). This hydrophobic core is typically flanked by a population of positively charged residues (Figure 1A). To date, thousands of single-site substitutions within the human IF protein sequences correlating with pathogenic syndromes or non-pathogenic phenotypes have been identified. The abundant single-site variation records provide us with an enriched database to test the selection and conservation of positively charged residues flanking the nonpolar domain.

Inspection of the IF database leads to an important observation that the occurrence of diseases associated with single cation-to-cation substitution, that is the substitutions among arginine (R), lysine (K), and histidine (H), exhibits a strong correlation with the positions flanking the nonpolar patch, i.e*.*, ***e*** and ***g*** (Figure 1B, Supplementary Table S1). We began with the point mutation at the “hotspot” R125 located in the α-helical 1A domain of keratin 14 (KRT14, type I IF family), where substitution of R125 by histidine leads to the occurrence of a monogenic disease, specifically the Dowling-Meara type of epidermolysis bullosa simplex (EBS) (Coulombe et al., 1991) (Figure 1C). This fact suggests that the identity of the positively charged residue at 125 in KRT14 is conserved to be arginine. The interchange between arginine and its positively charged homolog results in a disordered syndrome. We searched in the Human Intermediate Filament Database (http://www.interfil.org/index.php) (Szeverenyi et al., 2008) and screened the 28 members in the type I family (i.e., KRT9, 10, 12–20, 23–28, and 31–40). As revealed by the multiple-sequence alignment (MSA) algorithm created using ClustalW (Larkin et al., 2007) seen in Supplementary Figures S1A, 5 proteins (KRT14, 10, 13, 16, and 17) among the 28 type I members have been documented to carry a hotspot cationic residue at the analogous positions of R125 in KRT14, where a single site substitution among different cationic homologs (lysing, arginine, and histidine) occurring at this position leads to a pathogenic syndrome (Supplementary Figure S1B). Specifically, the pathogenic mutations include the R156H in KRT10 corresponding to epidermolytic hyperkeratosis (bullous congenital ichthyosiform erythroderma) (Cheng et al., 1992; Rothnagel et al., 1992), R114H in KRT13 corresponding to oral white sponge naevus (Nishizawa et al., 2008), R127H in KRT16 corresponding to pachyonychia congenital-K16 (Wilson et al., 2014), and R94H in KRT17 corresponding to pachyonychia congenital type 2 (Smith et al., 1997). Collective structural biology evidence illustrated the coiled-coil characteristic features of these IF proteins (Fuchs and Weber, 1994; Herrmann and Aebi, 2004; Parry, 2005; Kim and Coulombe, 2007). α-Helical heptads can be depicted as repeats of seven residues (*a-b-c-d-e-f-g*). As demonstrated by the helix-wheel diagrams in Supplementary Figure S1B, the five helical wheels of KRT 14, 10, 13, 16, and 17 exhibit the same hydrophobic regions (L, I, V, Y, W or M) at the positions ***a*** and ***d***, whereas the conserved arginine residues are located at the flanking position ***g***. This preliminary result conveys a message that the identity of proximal positive charged residues, R125 and its analogous sites, close to the hydrophobic domain are not interchangeable in the IF superfamily.

Inspired by the discovery of the conserved R125 in KRT 14 (named site I) and analogues, we screened the 4,999 disease-correlated mutations throughout the IF superfamily in the Human Intermediate Filament Database and identified an additional nine distinct sites, besides site I, in the six IF families bearing variations among positively charged residues which are related to human diseases (Figures 1D–L): site II (R388H in KRT14) is associated with epidermolysis bullosa simplex Weber-Cockayne type (EBS-WC) (Abu Sa’d et al., 2006); site III (K199R in KRT5) is associated with EBS-WC (Abu Sa’d et al., 2006); site IV (R471H in KRT5) is associated with EBS-WC (Wertheim-Tysarowska et al., 2016); site V (R341H in KRT8) is associated with liver disease, inflammatory bowel disease, and primary biliary cirrhosis (Ku et al., 2005); site VI (H326R in desmin) is associated with sudden cardiac death related disease (Brodehl et al., 2013); site VII (R79H in glial fibrillary acid protein, GFAP) is associated with Alexander disease (Brenner et al., 2001; Rodriguez et al., 2001); site VIII (R239H in GFAP) is associated with Alexander disease (Brenner et al., 2001; Rodriguez et al., 2001); site IX (R377H in lamin A) is associated with limb-girdle muscular dystrophy type 1B, dilated cardiomyopathy 1A, and Emery-Dreifuss muscular dystrophy (Muchir et al., 2000); and site X (R339H in phakinin) is associated with cataract 12 (Ma et al., 2008). These specific pathogenic mutations are located in the helical rod domain of the corresponding proteins. As illustrated by the helix-wheel diagrams in Figure 1, most pathogenic mutations (I–V and VII–X) occur at positions ***e*** or ***g*** in proximity to the hydrophobic regions at ***a*** and ***d***. A single exception, the H326R mutation in desmin (site VI), which barely impacts the folding and assembly structure of desmin, is at position ***f*** far from the continuous hydrophobic regions at ***a*** and ***d*** (Figure 1H) (Brodehl et al., 2013).

**FIGURE 2:**
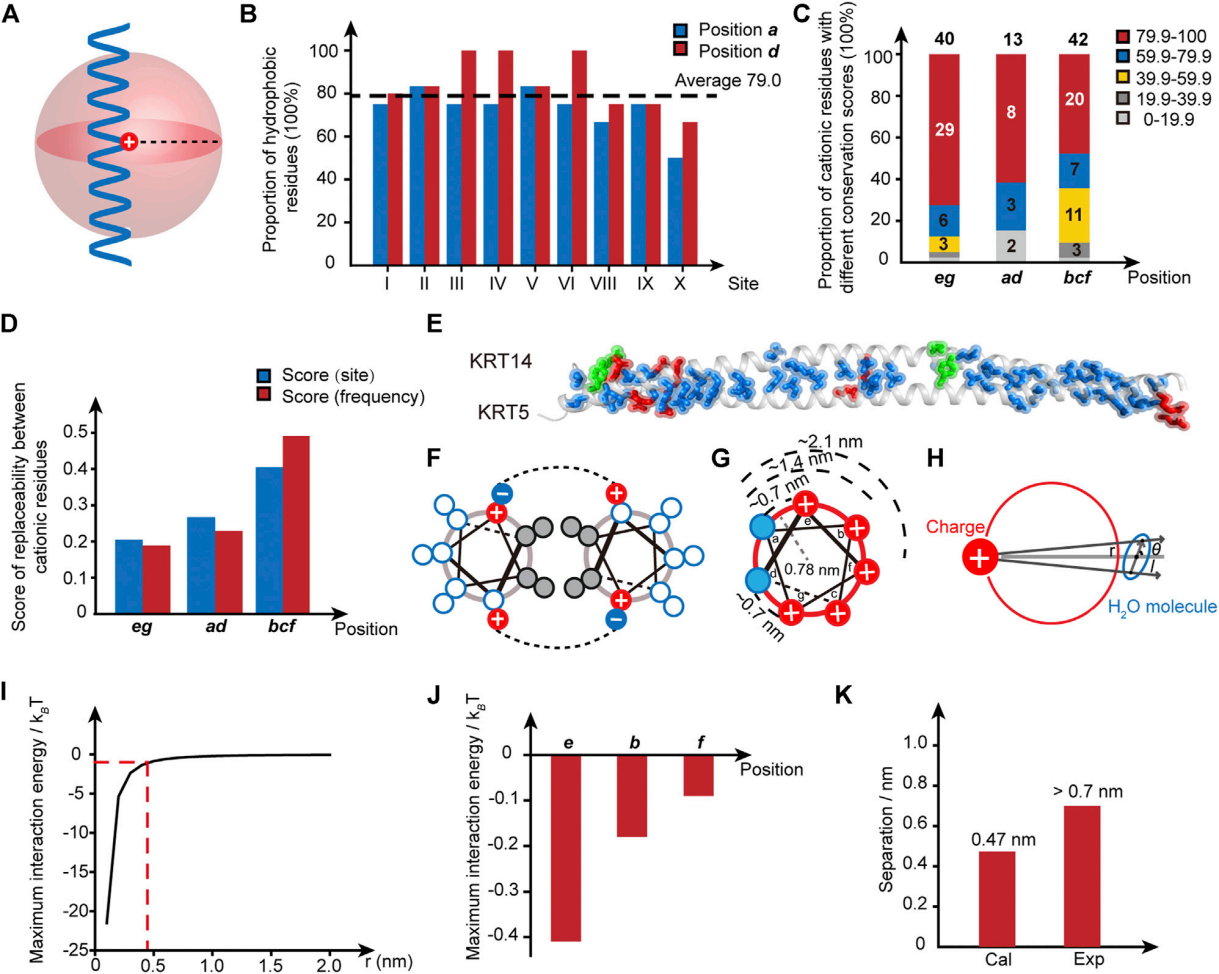
The identity of cationic sides proximal to the hydrophobic domain is conserved. **(A)** Schematic illustration of the local region surrounding the cationic pathogenic mutation sites in a helical rod domain. **(B)** The proportion of hydrophobic residues for the residues in lanes ***a*** and ***d*** that are close to the pathogenic mutation sites. The average score is indicated by the dotted line. Blue, position ***a***; red, position ***d***. **(C)** The conservation score of the positively charged residues for the positions ***eg***, ***ad***, and ***bcf***. Different conservation scores are encoded by different colors. The population of samples is indicated by the number in the column. **(D)** The scores of replaceability among different positively charged residues in the wild-type IF superfamily. Blue, the replaceability score counted by the number of homology sites involving the substitution among cationic amino acids; red, the replaceability score counted by the occurring frequency of cation-to-cation substitutions. **(E)** Diagram of the intermolecular interaction pairs formed in a coiled-coil architecture. KRT5-KRT14 coil 2B complex (PDB code: 3TNU) is shown as a representative example. Red, electrostatic interactions; blue, hydrophobic interactions; green, hydrogen bonds. **(F)** Helix-wheel diagram of a coiled-coil dimer and the intermolecular interactions. Grey, hydrophobic residues; red, positively charged residues; blue, negatively charged residues. **(G)** Schematic diagram of the distances between the cationic residues and the nonpolar patch. (**H**) Schematic illustration of the charge-dipole interaction between the cationic side chain and interfacial water. Red circle, the cross-section of a helix; solid red disk with a white plus sign, cationic side chain, blue sphere, interfacial water molecules. (**I**) The maximum charge-dipole interaction energy between the cationic side chain and the interfacial water is dependent on the separation between the charged group and the water. The dashed line is indicative of the separation of 0.47 nm when the maximum interaction energy is equal to 1 *k*_*B*_ *T*. (**J**) The maximum charge-dipole interaction energy calculated for the charged groups at the sites of ***e***, ***b***, and ***f***, respectively. (**K**) The separation with the calculated charge-dipole interaction energy of 1 *k*_*B*_ *T* is compared with the separation existing between the charged group and the interfacial water.

The similarity in the structural patterns exhibited by the disease-correlated sites (Figure 1) motivates us to analyze the pertinent properties of the local region surrounding the hotspot sites. Two conclusions can be drawn from the analysis. First, a hydrophobic patch close to the pathogenic mutation sites is consensual. For example, take the E115-K146 segment in KRT14 to analyze the hydrophobicity of the microenvironment surrounding site I, R125. In lane ***a***, there are four residues (M119, L126, V133, and N140) located within a separation distance of three sets of heptad repeats (21 residues, ∼ 3 nm, Figures 1C, 2A) from the conversed site R125 in the α-helical domain. Thus, the proportion of hydrophobic residues at this position is 75% (Figure 2B and Supplementary Table S2). Similarly, the proportion of hydrophobic residues in lane ***d*** is 80%. As shown in Figure 2B, hydrophobic residues dominate the proximity of 10 pathogenic mutation sites where the average proportion of hydrophobic residues in lanes ***a*** and ***d*** is 79%. Second, the conservation of the cationic residue identity displays a dependence on the relative position to the hydrophobic patch. To reflect the diversity of a specific cationic residue in the homology sequences within a wild-type human IF family, we defined a conservation score (*C*) as:

$$C=\frac{H}{T}\times 100\%$$ (1)

where *H* is the number of homology sequences carrying a conserved cationic residue at the analogous site, and *T* is the total number of homology sequences within the single-family in the MSA analysis. For instance, the analogous site of R125 in KRT14 is conserved through the members of the type I family in the MSA analysis (human KRT9-10, 12–20, 23–28, 31–32, 33A, 33 B, and 34) (Supplementary Figure S2A), and thus the conservation score of R125 is 100%. Whereas for the analogous site of K132 in KRT14, there is a total of 22 homologous members in the MSA analysis in which 16 members carry a lysine at this site. Correspondingly, the conservation score of K132 is 16/22 ≈ 72.7%. We calculated the conservation scores for all cationic residues in the mutation-located helical rod domains of each disease-causing sequence in the IF superfamily (Supplementary Table S3). Intriguingly, the percentage of positively charged residue with a high conservation score (>79.9%) decreases from 72.5% (29/40) at the positions ***e*** and ***g*** (proximal to the hydrophobic patch), to 61.5% (8/13) at the positions ***a*** and ***d*** (located in the hydrophobic patch), and to 47.6% (20/42) at the positions ***b***, ***c*** and ***f*** (far from the hydrophobic patch) (Figure 2C). The average conservation score of positively charged residue throughout the IF superfamily deceases from 84.3% (***e***, ***g***), to 80.5% (***a***, ***d***), and to 69.7% (***b***, ***c***, and ***f***) (Supplementary Table S3). This result suggests there exists an identity selection of the adjacent cationic residues flanking the hydrophobic domain of a helix and that these cationic residues are conserved in evolution. In contrast, the cationic residues that are not adjacent to the hydrophobic domains, i.e., the cations at the positions ***b***, ***c***, and ***f***, tolerate higher levels of variation and replaceability among different types of cationic side chains (Figure 2D and Supplementary Table S4).

We have shown that the identity of the flanking cationic residues around the hydrophobic patch is delicately selected by nature, i.e., the interchange among cationic residues at this position is rare in nature and is usually correlated with the occurrence of pathogenic syndromes. An important question emerging from this finding is why these conserved cations are located at the boundary of the hydrophobic domain. Considering there exist dozens of intermolecular interaction pairs (Figure 2E) between IF helices that maintain a coiled-coil, the fluctuation associated with the single-site substitution to the localized salt bridge formed between the cationic side chain on one helix and the anion side chain on another helix within a coiled-coil pair (Figures 2E,F) appears unlikely to account for a delocalized and long-distance perturbation in the architectures of IF proteins. Alternatively, the structural perturbations generated by the single-site mutation reflect the interplay between the proximal cationic side chains and the hydrophobic interactions mediated by the nonpolar patch. This aligns with our previous observation of the globally amphiphilic β-amino acid oligomer system (Ma et al., 2015; Wang et al., 2017; Wang et al., 2021). Specifically, the substitution among different types of cationic side chains perturbs the local structure and density of the interfacial water around the mutation site, affecting the hydration shell around the nonpolar domain and resulting in a fluctuation in the magnitude of hydrophobic interactions.

It is necessary to comment that whether the intermolecular network presented by IF proteins would adopt a similar modulation effect as the β-peptide is not obvious, considering the complexity of the chemical pattern and noncovalent network present on the native protein surface relative to the synthetic β-peptide. If such a mechanism does exist in the α-amino acid system, it coincides with the previous experimental and theoretical results that the effective length scale of the interfacial charge modulation effect on the interfacial water shell is around 1 nm (Chandler, 2005; Acharya et al., 2010; Wang et al., 2017). The cationic residues at positions ***e*** and ***g*** are ∼0.7 nm away from the nonpolar patch and thus are conserved to preserve the charge-related interactions and hydrophobic interactions of the structures (Figure 2G). In contrast, the cationic residues at positions ***b***, ***f***, and ***c*** are too distant (the distance between ***a*** and ***b*** is ∼1.4 nm, and the distance between ***a*** and ***f*** is ∼2.1 nm) to impact the hydrophobic interactions mediated by the nonpolar domain, and thus they display a higher level of mutation tolerance (the conservation degree of cationic residues at positions ***a*** and ***d*** is discussed in the SI). Two mechanisms might be useful in the understanding of the effective length scale. The first mechanism is the charge-dipole interaction occurring between the cationic side chain and the interfacial water molecules near the nonpolar patch. The second mechanism is proposed to originate from the unique organizational structure and density of the interfacial water molecules surrounding the mutation site, as the perturbation of the organized water structure caused by the interface usually decays within 1 nm of the interface (Bjorneholm et al., 2016).

To provide insight into the mechanism corresponding to the conversation of cationic residues at positions ***e*** and ***g***, we calculated the interaction energy *W*(*r*, *θ*) for a charged side chain interacting with water as (Figure 2H):

$$W\left(r,\theta \right)=-\frac{Qq}{4\pi \varepsilon_{0}\varepsilon }\left[\frac{1}{r-\frac{1}{2}lcos\theta }-\frac{1}{r+\frac{1}{2}lcos\theta }\right]$$ (2)

where *Q* is the point-charge carried by the cationic side chain, q is the dipole charge carried by the water, *r* is the separation between the water and the charged group, *l* is the water dipole length, *ε*_*0*_ is the vacuum permittivity, *ε* is the permittivity of a helix (approximately equal to the value for oil at ∼10), and *θ* is the included angle between the molecular axis of water and the line connecting the charged side chain and the water. When *θ* is equal to 0, the dependence of the maximum interaction energy, *W*(*r*, *θ* = 0), at 300 K on the separation between the cationic side chain and the water can be calculated (Figure 2I). As revealed by this calculation, the magnitudes of the charge-dipole interactions for the cationic residues at positions ***e***, ***b***, and ***f*** are determined to be less than 1 *k*_*B*_ *T*, which is too small to resist thermal motion (Figure 2J). The divergence between the separation distance (0.47 nm) corresponding to the calculated charge-dipole interaction of 1 *k*_*B*_ *T* and the effective separation distance (>0.7 nm) measured from the protein database (Figure 2K) hints that the high conservation degree of cationic residues at positions ***e*** and ***g*** within the IF superfamily is correlated with the perturbation of interfacial water molecules by the cationic side chain instead of charge-dipole interactions.

We end this paper by making two additional comments. First, as an alternative pathogenic mechanism, post-translational modifications of cationic side chains potentially play a role in the disease development associated with the single cation-to-cation substitution within the IF superfamily. For example, K residues, instead of R residues, in the keratins can be modified by acetylation, ubiquitination, SUMOylation, and transamidation (Jacob et al., 2018). These post-translational modifications presumably provide a hierarchical regulation of the keratin self-association (Jacob et al., 2018). Second, the pKa of H side chains in a protein is typically below or near 7 (Grimsley et al., 2009), resulting in a partial deprotonation of the H side chain to some extent in a normal physiological environment, which is in contrast to the full protonation of K and R side chains in a protein (Kubyshkin, 2021).

As a summary, we traversed the disease-correlated mutations occurring in the coiled-coil IF superfamily and identified 10 distinct hotspots that carry a pathogenic cation-to-cation substitution. The chemical diversity of these hotspots is positively dependent on the separation distance between the cationic site and the nonpolar patch. Specifically, the cationic residues which flank the nonpolar patch are relatively conserved compared to those that are distant from the nonpolar patch. Considering the prevalence of the coiled-coil supramolecular structure, involved in 5.8% of the protein structures documented in the protein data bank (PDB), the importance of our results is not limited to the understanding of IF protein assembly structures but extends to a broad perspective for revealing the interplay among the pairwise noncovalent interactions in biologically relevant systems (Hadley et al., 2008; Wang et al., 2017). In the future, more experimental evidence and theoretical calculations may be needed to elucidate the effects of the flanking charged group on the hydrophobically driven folding and assembly in native protein systems. This effort will greatly leverage our understanding of design principles that can harness these intermolecular interactions and may offer a promising, rational design method for diverse supramolecular systems. In particular, the discovery of the strong impact that the nanopatterning of hydrophobic and cationic groups exerts on the self-assembly of artificial and natural systems could have an enormous impact on a variety of research fields relevant to supramolecular chemistry, such as the *de novo* calculation of protein folding and association, drug delivery platforms, and the design of stimuli-responsive materials.

## Data Availability

The original contributions presented in the study are included in the article/Supplementary Material, further inquiries can be directed to the corresponding authors.
