# Supplementary material for: Conservation and Identity Selection of Cationic Residues Flanking the Hydrophobic Regions in Intermediate Filament Superfamily
Source: Front Chem. 2021 Sep 2;9:752630. doi: 10.3389/fchem.2021.752630 (PMC8443778; doi:10.3389/fchem.2021.752630)
Supplement: Supplementary file 1 [file Presentation1.PDF]

Supplementary Material

Conservation and Identity Selection of Cationic Residues Flanking the Hydrophobic Regions in Intermediate Filament Superfamily

Wenbo Zhang[1†], Mingwei Liu[1†], Robert L. Dupont[2], Kai Huang[3], Lanlan Yu[1], Shuli Liu[4], Xiaoguang Wang[2,5*], Chenxuan Wang[1*]

[1]State Key Laboratory of Medical Molecular Biology, Institute of Basic Medical Sciences Chinese Academy of Medical Sciences, School of Basic Medicine Peking Union Medical College, Beijing 100005, China

[2]William G. Lowrie Department of Chemical and Biomolecular Engineering, The Ohio State University, Columbus, OH 43210, USA

[3]Shenzhen Bay Laboratory, Shenzhen 518118, China

[4]Department of Clinical Laboratory, Peking University Civil Aviation School of Clinical Medicine, Beijing 100123, China

[5]Sustainability Institute, The Ohio State University, Columbus, OH 43210, USA

†Co-first authors

*** Correspondence:**
Prof. Chenxuan Wang, email: wangcx@ibms.pumc.edu.cn

Prof. Xiaoguang Wang, email: wang.12206@osu.edu

The conservation degree of the cationic residues buried by the nonpolar region

The cationic residues at positions ***a*** and ***d***, which are also surrounded by nonpolar residues, exhibit a high average conservation score (80.5%) comparable with the cationic residues at the positions ***e*** and ***g*** (84.3%) (Table S3). The cations at positions ***a*** and ***d*** are buried to form an intermolecular interface between multiple α-helices and crucial for modulating the oligomeric state of IF protein[1-5]. Conventionally, the role of the cationic residues at positions ***a*** and ***d*** are attributed to the hydrogen bonds across the interface between helices or achieve the complementarity in side chain packing[1-4]. The understanding of the potential impact of the adjacent cation on hydrophobic interactions provides us with a fresh perspective that the cationic side chains at positions ***a*** and ***d*** might participate in the modulation of the hydrophobic interactions encoded by IF proteins. It is noteworthy that positions ***a*** and ***d*** are not associated with the highest conservation score, although they are in direct contact with the hydrophobic patch. This observation reminds us of the complexity of the interplay between the hydrophobic interactions and the charge-related effects. Previous molecular dynamics simulations of β-peptide suggest that embedded charges are more influential than proximal ones in regulating hydrophobic interactions[6]. Hydrophobic surfaces can also enhance nearby electrostatic interaction by reducing the local dielectric constant of water[7,8]. However, one should keep in mind that a hydrophobic environment can shift the local chemical equilibrium of ionization to favor the neutralization of charges

via charge regulation[9]. Taking such charge regulation mechanism into account, the impact of charges on hydrophobic interaction would be not only dependent on their distances to the hydrophobic domain but also their modulated effective pK_a.

References:

(1) Deng, Y.; Liu, J.; Zheng, Q.; Eliezer, D.; Kallenbach, N. R.; Lu, M. *Structure* **2006**, *14*, 247.
(2) Liu, J.; Zheng, Q.; Deng, Y.; Cheng, C. S.; Kallenbach, N. R.; Lu, M. *Proc. Natl. Acad. Sci. U. S. A.* **2006**, *103*, 15457.
(3) Hadley, E. B.; Testa, O. D.; Woolfson, D. N.; Gellman, S. H. *Proc. Natl. Acad. Sci. U. S. A.* **2008**, *105*, 530.
(4) Akey, D. L.; Malashkevich, V. N.; Kim, P. S. *Biochemistry* **2001**, *40*, 6352.
(5) Gonzalez, L., Jr.; Woolfson, D. N.; Alber, T. *Nat. Struct. Biol.* **1996**, *3*, 1011.
(6) Huang, K.; Gast, S.; Ma, C. D.; Abbott, N. L.; Szlufarska, I. *J. Phys. Chem. B* **2015**, *119*, 13152.
(7) Chen, S.; Itoh, Y.; Masuda, T.; Shimizu, S.; Zhao, J.; Ma, J.; Nakamura, S.; Okuro, K.; Noguchi, H.; Uosaki, K.; Aida, T. *Science* **2015**, *348*, 555.
(8) Sato, T.; Sasaki, T.; Ohnuki, J.; Umezawa, K.; Takano, M. *Phys. Rev. Lett.* **2018**, *121*, 206002.
(9) Huang, K.; Szleifer, I. *J. Am. Chem. Soc.* **2017**, *139*, 6422.

Table S1. The diseases associated with single cation-to-cation substitution in IF superfamily

<table>
<tr><th>SITE</th><th colspan="5">IF/Disease</th><th>position</th></tr>
<tr><td rowspan="2">Ⅰ</td><td>KRT14</td><td>KRT10</td><td>KRT13</td><td>KRT16</td><td>KRT17</td><td rowspan="2">g</td></tr>
<tr><td>epidermolysis bullosa simplex-DM</td><td>epidermolytic hyperkeratosis</td><td>oral white sponge naevus</td><td>pachyonychia congenital-K16</td><td>pachyonychia congenital-type 2</td></tr>
<tr><td rowspan="2">Ⅱ</td><td colspan="5">KRT14</td><td rowspan="2">e</td></tr>
<tr><td colspan="5">epidermolysis bullosa simplex-WC</td></tr>
<tr><td rowspan="2">Ⅲ</td><td colspan="5">KRT5</td><td rowspan="2">g</td></tr>
<tr><td colspan="5">epidermolysis bullosa simplex-WC</td></tr>
<tr><td rowspan="2">Ⅳ</td><td colspan="5">KRT5</td><td rowspan="2">e</td></tr>
<tr><td colspan="5">epidermolysis bullosa simplex-WC</td></tr>
<tr><td rowspan="2">Ⅴ</td><td colspan="5">KRT8</td><td rowspan="2">g</td></tr>
<tr><td>liver disease</td><td colspan="3">inflammatory bowel disease</td><td>primary biliary cirrhosis</td></tr>
<tr><td rowspan="2">Ⅵ</td><td colspan="5">Desmin</td><td rowspan="2">f</td></tr>
<tr><td colspan="5">sudden cardiac death related disease</td></tr>
<tr><td rowspan="2">Ⅶ</td><td colspan="5">GFAP</td><td rowspan="2">g</td></tr>
<tr><td colspan="5">Alexander disease</td></tr>
<tr><td rowspan="2">Ⅷ</td><td colspan="5">GFAP</td><td rowspan="2">e</td></tr>
<tr><td colspan="5">Alexander disease</td></tr>
<tr><td rowspan="2">Ⅸ</td><td colspan="5">LaminA</td><td rowspan="2">e</td></tr>
<tr><td colspan="2">limb-girdle muscular dystrophy type 1B,</td><td colspan="2">dilated cardiomyopathy 1A</td><td>Emery-Dreifuss muscular dystrophy</td></tr>
<tr><td rowspan="2">Ⅹ</td><td colspan="5">Phakinin</td><td rowspan="2">e</td></tr>
<tr><td colspan="5">cataract 12</td></tr>
</table>

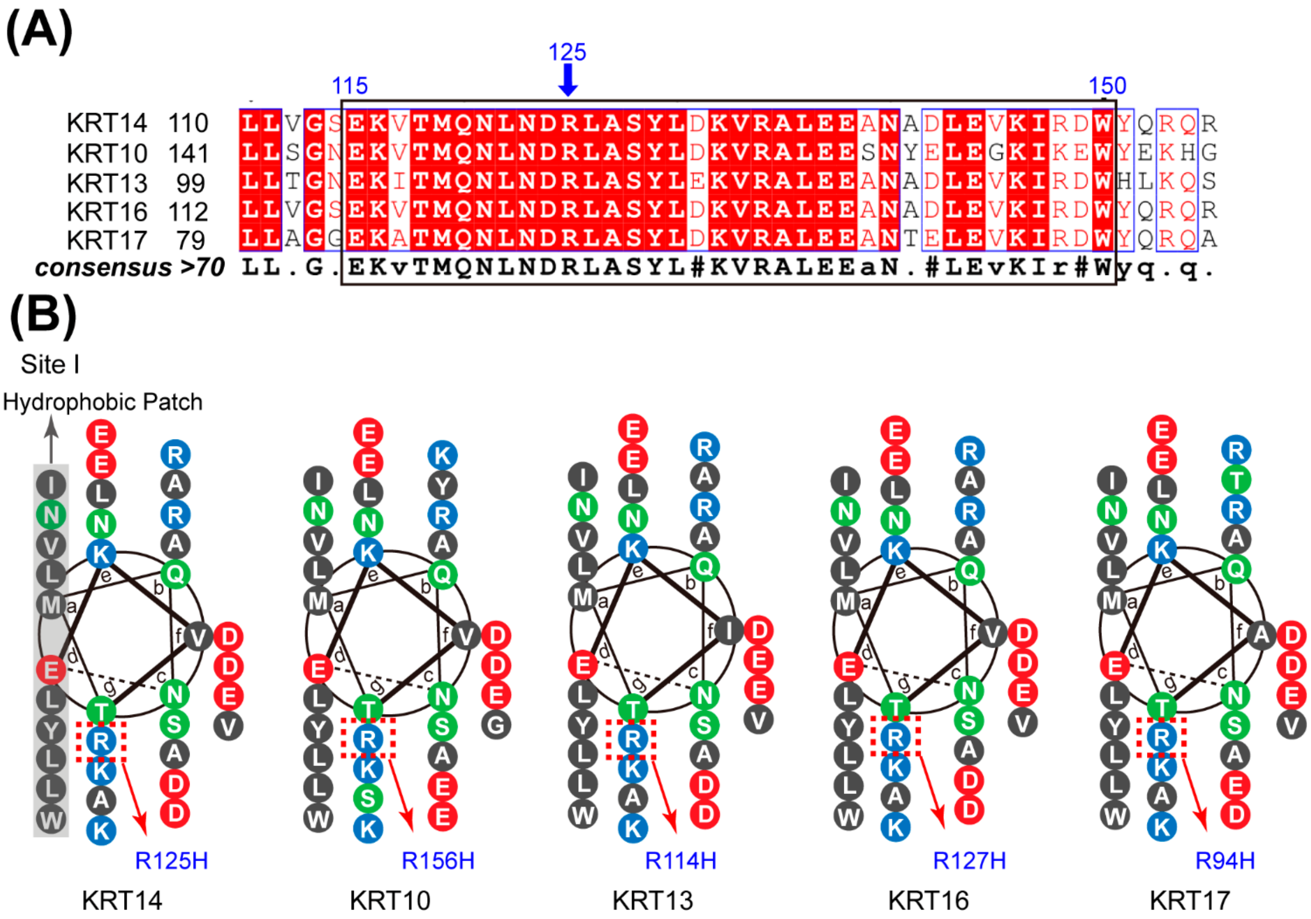

Figure S1. Multiple-sequence alignment and the helix-wheel diagrams of KRT14, 10, 13, 16, and 17 in type I family. (A) The multiple-sequence alignment of KRT14, 10, 13, 16, and 17 from *H. sapiens*. Segments that adopt an α-helical conformation in coil 1A domain (UniprotKB database was referenced) are indicated by the black box. The boundaries of the helical domain are labeled in blue (E115 and W150 in KRT14) and the "hotspot" arginine (R125 in KRT14) is indicated by arrows. The beginning residues of each sequence are labeled behind the protein names. The consensus sequence is added in the alignment figure. **(B)** Helix-wheel diagrams of the coil 1A domains in KRT14, 10, 13, 16, and 17. Blue, cationic residues; red, anionic residues; black, nonpolar residues; green, polar and uncharged residues. The hotspot R125 and its analogous sites in the homology sequences are labeled by red dotted boxes.

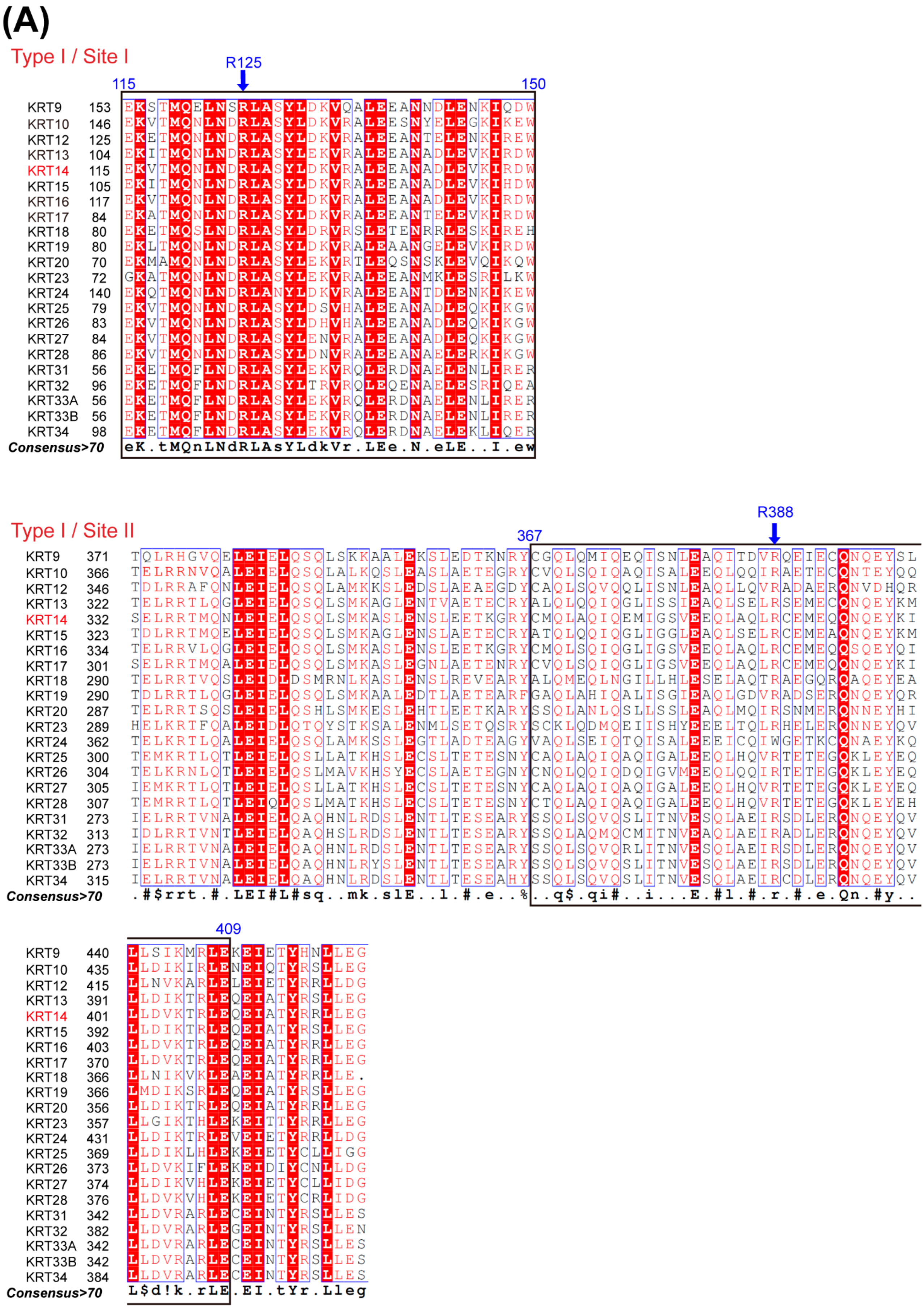
(A)
Type I / Site I
R125
115
150
Consensus>70
Type I / Site II
R388
367
409

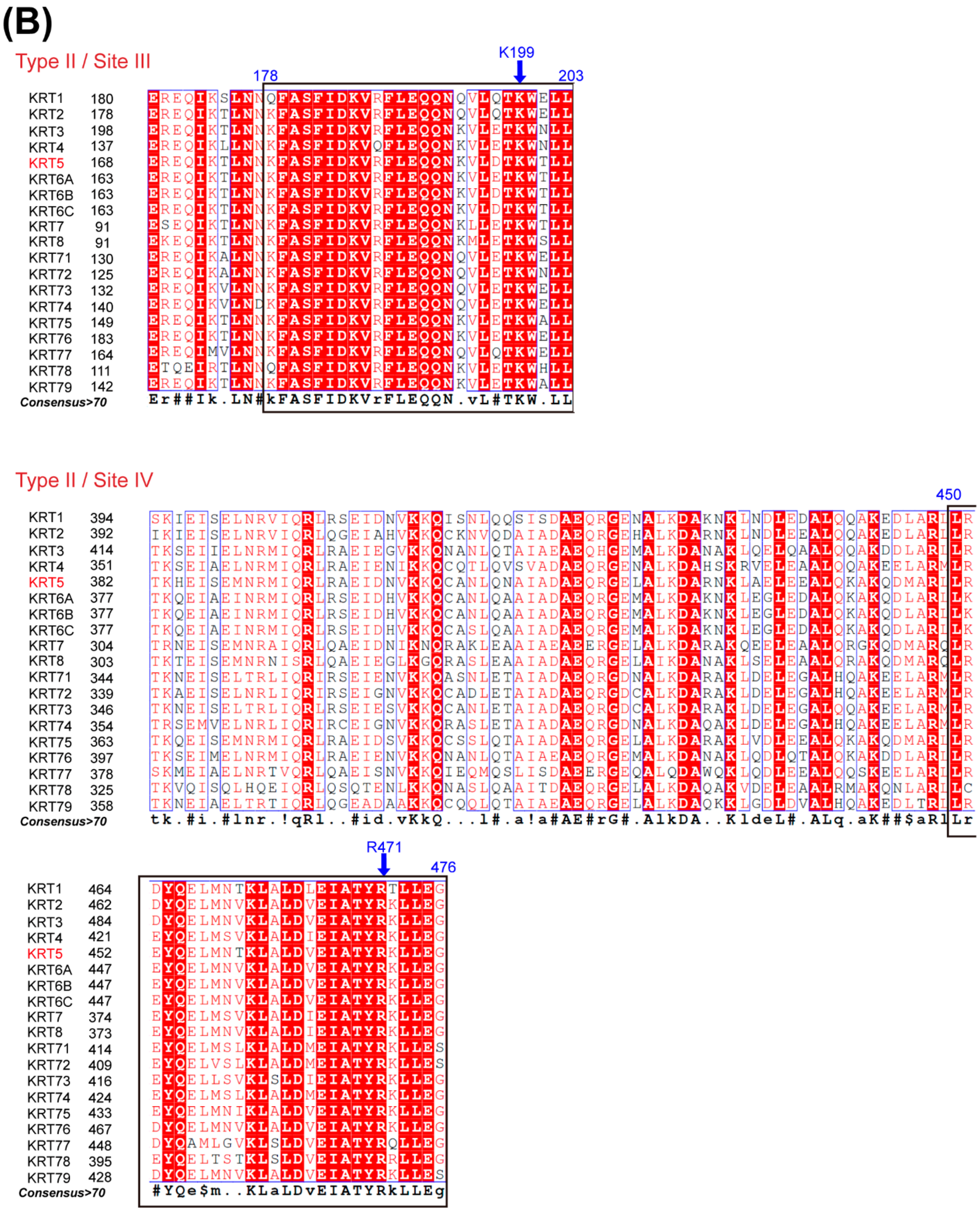
(B)
Type II / Site III
K199
178
203
KRT1 180
KRT2 178
KRT3 198
KRT4 137
KRT5 168
KRT6A 163
KRT6B 163
KRT6C 163
KRT7 91
KRT8 91
KRT71 130
KRT72 125
KRT73 132
KRT74 140
KRT75 149
KRT76 183
KRT77 164
KRT78 111
KRT79 142
Consensus>70
Type II / Site IV
450
KRT1 394
KRT2 392
KRT3 414
KRT4 351
KRT5 382
KRT6A 377
KRT6B 377
KRT6C 377
KRT7 304
KRT8 303
KRT71 344
KRT72 339
KRT73 346
KRT74 354
KRT75 363
KRT76 397
KRT77 378
KRT78 325
KRT79 358
Consensus>70
R471
476
KRT1 464
KRT2 462
KRT3 484
KRT4 421
KRT5 452
KRT6A 447
KRT6B 447
KRT6C 447
KRT7 374
KRT8 373
KRT71 414
KRT72 409
KRT73 416
KRT74 424
KRT75 433
KRT76 467
KRT77 448
KRT78 395
KRT79 428
Consensus>70

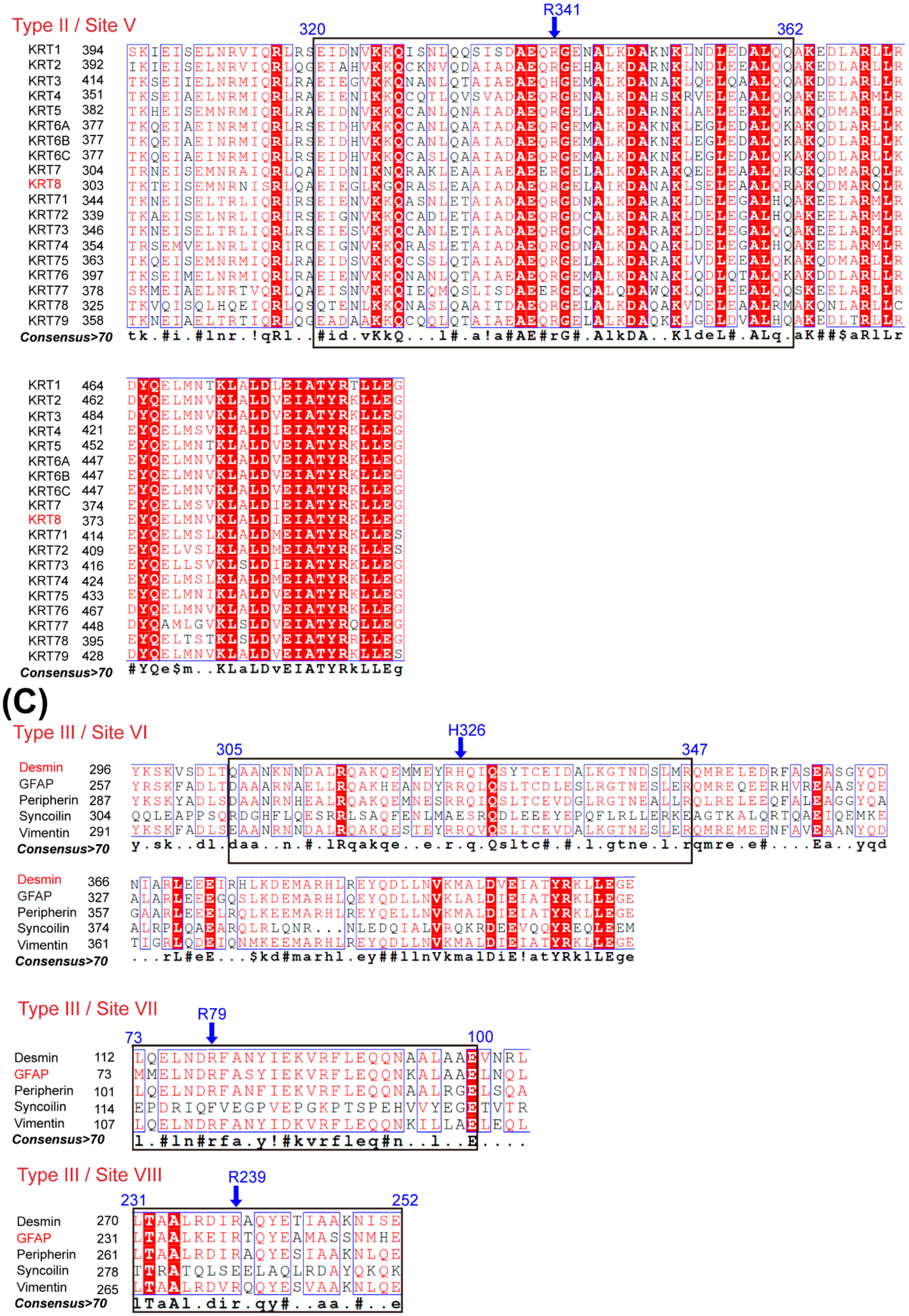
Type II / Site V
R341
320
362
KRT1 394
KRT2 392
KRT3 414
KRT4 351
KRT5 382
KRT6A 377
KRT6B 377
KRT6C 377
KRT7 304
KRT8 303
KRT71 344
KRT72 339
KRT73 346
KRT74 354
KRT75 363
KRT76 397
KRT77 378
KRT78 325
KRT79 358
Consensus>70
KRT1 464
KRT2 462
KRT3 484
KRT4 421
KRT5 452
KRT6A 447
KRT6B 447
KRT6C 447
KRT7 374
KRT8 373
KRT71 414
KRT72 409
KRT73 416
KRT74 424
KRT75 433
KRT76 467
KRT77 448
KRT78 395
KRT79 428
Consensus>70
(C)
Type III / Site VI
H326
305
347
Desmin 296
GFAP 257
Peripherin 287
Syncoilin 304
Vimentin 291
Consensus>70
Desmin 366
GFAP 327
Peripherin 357
Syncoilin 374
Vimentin 361
Consensus>70
Type III / Site VII
R79
73
100
Desmin 112
GFAP 73
Peripherin 101
Syncoilin 114
Vimentin 107
Consensus>70
Type III / Site VIII
R239
231
252
Desmin 270
GFAP 231
Peripherin 261
Syncoilin 278
Vimentin 265
Consensus>70

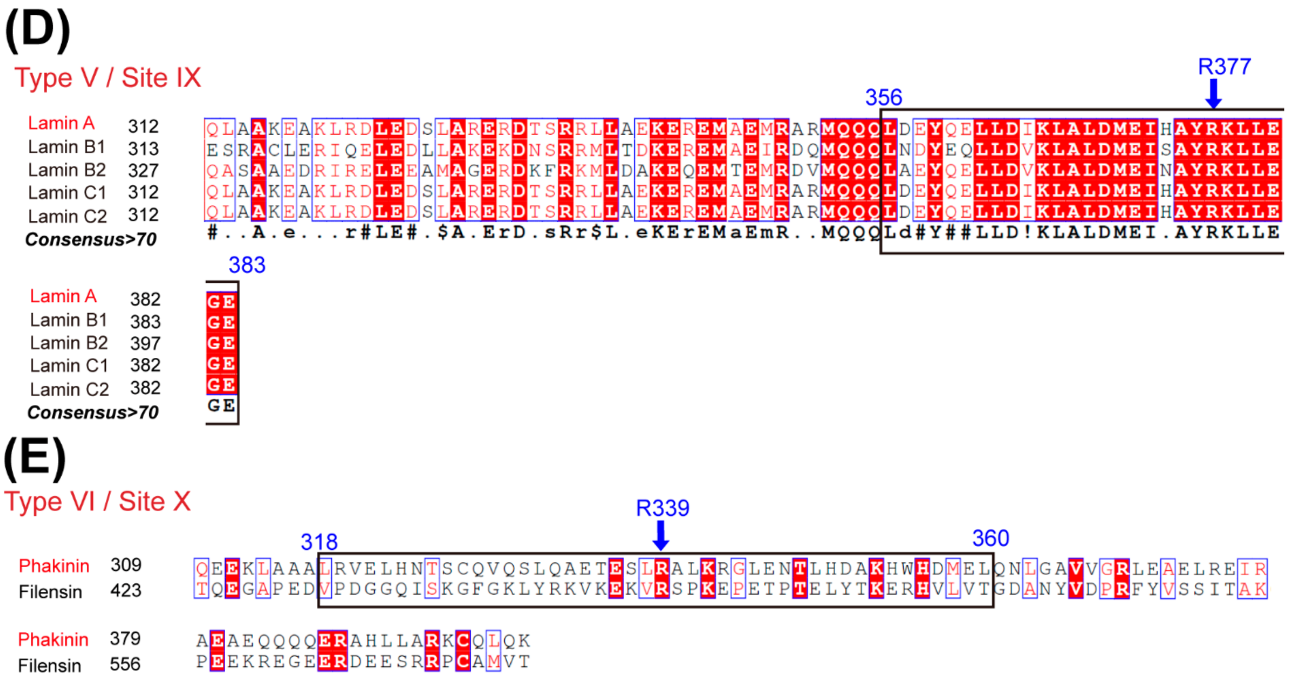

Figure S2. Multiple-sequence alignment of the helical domain regions containing a pathological mutated residue from the type I family (Site I and II) (A), type II family (Site III, IV, and V) (B), type III family (Site VI, VII, and VIII) (C), type V family (Site IX) (D), and type VI family (Site X) (E). The beginning residues of each sequence are labeled behind the protein names. The pathological mutated residues in specific proteins (labeled in red) are indicated by blue arrows. Segments that adopt an α-helical conformation as shown in the helix-wheel diagrams in Figure 3 are indicated by black rectangular boxes. The boundaries are labeled in blue according to the specific protein labeled in red. The two boundaries of the helical domains are determined based on the following information: ① Site I: coil 1A (E115-W150) in KRT14 referred to the annotation of P02533 in UniprotKB database. ② Site II: coil 2B (S332-G421) in KRT14 referred to the KRT5/KRT14 heterodimer crystal structure (PDB code:3TNU). ③ Site III: coil 1A (E168-L203) in KRT5 referred to the annotation of P13647 in the UniprotKB database. ④ Site IV: coil 2B (T382-G476) in KRT5 referred to the KRT5/KRT14 heterodimer crystal structure (PDB code:3TNU). ⑤ Site V: coil 2B (T303-G397) in KRT8 referred to the MSA with KRT5 according to the 3TNU crystal structure. ⑥ Site VI: coil 2B (Y296-E412) in Desmin referred to the annotation of P17661 in the UniprotKB database. ⑦ Site VII: coil 1A (M73-L104) in GFAP referred to the annotation of P14136 in the UniprotKB database. ⑧ Site VIII: coil 2A (L231-E252) in GFAP referred to the annotation of P14136 in the UniprotKB database. ⑨ Site IX: coil 2B (Q312-E383) in Lamin A referred to its crystal structure (PDB code: 1X8Y). ⑩ Site X: coil 2B (Q309-K400) in Phakinin referred to the annotation of P13515 in the UniprotKB database. The number of sequences in the MSA analysis is limited by the ClustalW software (max number of sequences = 30; max total length of sequences = 10000).

Table S2. The proportion of hydrophobic residues

		The proportion of hydrophobic amino acids (%) [a]	
Type I	Site I [b]	Position ***a***	3/4 = 75 [c]
		Position ***d***	4/5 = 80
	Site II	Position ***a***	5/6 = 83.3
		Position ***d***	5/6 = 83.3
Type II	Site III	Position ***a***	3/4 = 75
		Position ***d***	4/4 = 100
	Site IV	Position ***a***	3/4 = 75
		Position ***d***	3/3 = 100
	Site V	Position ***a***	5/6 = 83.3
		Position ***d***	5/6 = 83.3
Type III	Site VII	Position ***a***	3/4 = 75
		Position ***d***	4/4 = 100
	Site VIII	Position ***a***	2/3 = 66.7
		Position ***d***	3/4 = 75
Type V	Site IX	Position ***a***	3/4 = 75
		Position ***d***	3/4 = 75
Type VI	Site X	Position ***a***	3/6 = 50
		Position ***d***	4/6 = 66.7
Average	79.0		

[a] The residues that are located in the α-helical domain and within a separation distance of 3 times of heptad repeats (21 residues, ~ 3 nm) from the conserved site are counted.

[b] The exception site VI (H326R in desmin) is not included in the Table.

[c] Proportion of hydrophobic amino acids = number of hydrophobic residues/ total number of residues in a specific lane × 100%

Table S3. Conservation score of positively charged residues [a]

	Position ***e***		Position ***g***		Position ***a***		Position ***d***		Position ***b***		Position ***c***		Position ***f***	
Site I [b]	K116	100[c]	R125<br>K132<br>K146	100<br>72.7<br>68.2	N/A[e]		N/A		R134<br>R148	86.4<br>45.5	N/A		N/A	
Site II	R388<br>R416	95.5<br>77.3	N/A		R335<br>K363<br>K405	77.3<br>77.3<br>18.2	K352	77.3	R336<br>K399	95.5<br>22.7	R365<br>R407	40.9<br>68.2	R417	72.7
Site III	R169	84.2	K178<br>K185<br>K199	89.5<br>100<br>100	N/A		N/A		K173<br>R187<br>K194	84.2<br>94.7<br>63.2	N/A		N/A	
Site IV	K383<br>R397<br>K404<br>R429<br>K443<br>R471	100<br>68.4<br>100<br>36.8<br>100<br>100	R420<br>K431	94.7<br>100	K460	100	N/A		K426	94.7	R395<br>K441<br>R448	100<br>31.6<br>100	H384<br>R391<br>K405<br>R451<br>K472	5.3<br>94.7<br>89.5<br>78.9<br>84.2
Site V	K304<br>K325<br>K381	89.5<br>100<br>100	R341<br>R362<br>R368	94.7<br>10.5<br>100	R321	15.8	K352	100	K364<br>R392	100<br>100	R316<br>R372<br>K393	100<br>78.9<br>84.2	R312<br>K347	94.7<br>94.7
Site VII [d]	N/A		R79<br>K86	80<br>80	N/A		N/A		R88<br>K95	80<br>40	N/A		N/A	
Site VIII	R239	80	N/A		N/A		N/A		K236	20	N/A		N/A	
Site IX	R321<br>R335<br>R349<br>R377	80<br>100<br>100<br>100	K316<br>R351	60<br>60	R331<br>K366	80<br>100	K341	100			K319	60	R329<br>R336<br>R343<br>K378	60<br>80<br>80<br>100
Site X	R318<br>R339<br>K353<br>R388<br>K395	50<br>100<br>100<br>100<br>50	R369<br>H390	100<br>50	K342<br>H356	100<br>100	R394	100	H322<br>R343<br>H350<br>R378	50<br>50<br>50<br>50	K312<br>H354<br>R375	50<br>50<br>50	K400	50
SCORE	84.3				80.5				69.7					

[a] All positively charged residues in the helical domains (Fig. S1) are counted.

[b] Positively charged residues in KRT14 are regarded as representatives in the type I family.

[c] Conservation score (colored red) = the number of homology sequences carrying a specific cationic residue at the analogous site/the total number of homology sequences within the one family × 100 (%)

[d] The exception site VI (H326R in desmin) is not included in the Table.

[e] There are no positively charged residues at this position. The score is not applicable.

Table S4. The replaceability among positively charged residues [a]

		Position ***e*** and ***g***			Position ***a*** and ***d***			Position ***b***, ***c***, and ***f***		
Subfamily	Sites	Score (site) [b]	Residues	Score (frequency) [c]	Score (site)	Residues	Score (frequency)	Score (site)	Residues	Score (frequency)
Type I	Site I	2/4	K132	3/22	N/A[d]			2/2	R134	2/22
			K146	2/22					R148	8/22
	Site II	1/2	R416	1/22	3/4	R335	5/22	4/5	R336	1/22
						K352	5/22		R365	1/22
						K405	5/22		K399	1/22
									R407	5/22
	Score [f]	3/6	5/22		3/4	10/22		6/7	12/22	
Type II	Site III	1/4	R169	1/19	N/A			1/3	K173	1/19
	Site IV	2/8	K383	2/19	0/1	0/19		2/9	R451	3/19
			R420	1/19					K472	1/19
	Site V	2/5	K304	2/19	0/4	0/19		2/7	R372	3/19
			R341	1/19					K393	1/19
	Score	5/17	5/19		0/5	0/19		5/19	7/19	
Type III	Site VII [e]	0/2	0/5		N/A			1/2	R88	1/5
	Site VIII	0/1	0/5		N/A			1/1	K236	3/5
	Score	0/3	0/5					2/3	4/5	
Type V	Site IX	0/6	0/5		1/3	R331	1/5	3/5	K319	2/5
									R329	1/5
									R336	1/5
	Score	0/6	0/5		1/3	1/5		3/5	2/5	
Type X	Site X	0/7	0/2		0/3	0/2		1/8	R378	1/2
	Score	0/7	0/2		0/3	0/2		1/8	1/2	
Total score [g]		8/39 = 0.205	10/53 = 0.189		4/15 = 0.267	11/48 = 0.229		17/42 = 0.405	26/53 = 0.491	

[a] All positively charged residues in the helical domains (Fig. S1) are counted.

[b] The replaceability score (blue) is counted by the number of homology sites involving the substitution among cationic amino acids. For the same wild-type human family in the MSA analysis, take the homology sequence in which the presence of single cation-to-cation substitution is associated with the disease as the reference.

$$Score\ (site) = \frac{\text{the number of cationic residue sites occurring the substitution among cationic amino acids}}{\text{the total number of cationic residue sites in the specific positions in the wild−type human IF proteins}}$$

For example, there are 22 homology sequences in the MSA analysis for the site I type I family. Take the KRT14 E115-W150 segment as a reference. For the E115-W150 segment in KRT14, 4 cationic residues are occurring at the positions $\boldsymbol{e}$ and $\boldsymbol{g}$, *i.e.*, K116, R125, K132, and K146. Among these 4 cationic residues, K132 and K146 are observed to be substituted by other cationic amino acids in the homology sequences from the same type I subfamily in the MSA analysis. Thus, the score is 2/4.

[c] The replaceability score (red) is counted by the occurring frequency of cation-to-cation substitutions. For the same wild-type human family in the MSA analysis, the ratio of the number of cation-to-cation substitution at a specific homology site to the total number of homology sequences within one family.

$$Score\ (frequency) = \frac{\text{the number of substitutions among cationic amino acids}}{\text{the total number of homology sequences in the wild−type human IF proteins.}}$$

For example, among the 22 homology sequences of the E115-W150 segment of KRT14 in the MSA analysis, 15 sequences present a K, 2 sequences present an R, 4 sequences present an L, and 1 sequence presents a Q at the homology site of K146 in KRT14. Thus, for the homology site of K146 in KRT14, the frequency score of occurring cation-to-cation substitution is 2/22 (the frequency of K>R substitution). Similarly, 16 sequences are carrying a K, 2 sequences carrying an R, 1 sequence carrying 1 H, 2 sequences carrying a W, and 1 sequence carrying an S at the homology site of K132 in KRT14 in the MSA. Thus, for the homology site of K132 in KRT14, the frequency score of occurring cation-to-cation substitution is 3/22 (the frequency of K>R and K>H substitutions).

[d] There is no positively charged residues at this position. The score is not applicable.

[e] The exception site VI (H326R in desmin) is not included in the Table.

[f] The score for a specific family (shading in blue).

Score = the sum of the numerator in the specific position for each family / the sum of the denominator in the specific position for each family.

The repeated sequences have been removed.

[g] The total score for the whole IF superfamily (shading in red).

Total score = the sum of the numerator in the specific position for the whole IF superfamily / the sum of the denominator in the specific position for the whole IF superfamily.

These scores are positively correlated with the replaceability of cationic residue.
